# A Review of Nutrition Support Guidelines for Individuals with or Recovering from COVID-19 in the Community

**DOI:** 10.3390/nu12113230

**Published:** 2020-10-22

**Authors:** Abbie L. Cawood, Emily R. Walters, Trevor R. Smith, Rachel H. Sipaul, Rebecca J. Stratton

**Affiliations:** 1 Institute of Human Nutrition, Faculty of Medicine, Mailpoint 113, Southampton General Hospital, Tremona Road, Southampton SO16 6YD, UK; R.J.Stratton@soton.ac.uk; 2 Faculty of Health Sciences, University of Southampton, University Road, Southampton SO17 1BJ, UK; erw2v07@soton.ac.uk; 3 Department of Gastroenterology, Mailpoint 255, University Hospitals Southampton NHS Foundation Trust, Southampton General Hospital, Tremona Road, Southampton SO16 6YD, UK; trevor.smith@uhs.nhs.uk; 4 Consultant Dietitian, Southampton SO53, UK; rachel.sipaul@me.com

**Keywords:** COVID-19, malnutrition, nutrition support, recovery, rehabilitation, community

## Abstract

COVID-19 negatively impacts nutritional status and as such identification of nutritional risk and consideration of the need for nutrition support should be fundamental in this patient group. In recent months, clinical nutrition professional organisations across the world have published nutrition support recommendations for health care professionals. This review summarises key themes of those publications linked to nutrition support of adults with or recovering from COVID-19 outside of hospital. Using our search criteria, 15 publications were identified from electronic databases and websites of clinical nutrition professional organisations, worldwide up to 19th June 2020. The key themes across these publications included the importance in the community setting of: (i) screening for malnutrition, which can be achieved by remote consultation; (ii) care plans with appropriate nutrition support, which may include food based strategies, oral nutritional supplements and referral to a dietitian; (iii) continuity of nutritional care between settings including rapid communication at discharge of malnutrition risk and requirements for ongoing nutrition support. These themes, and indeed the importance of nutritional care, are fundamental and should be integrated into pathways for the rehabilitation of patients recovering from COVID-19.

## 1. Introduction

The COVID-19 pandemic has led to a rapid global response in the sharing of evidence, experience and best practice, perhaps in such a way we have never seen before. Professional organisations are contributing their collective expertise, enabling evidence-based guidance and pathways to be designed for patients with COVID-19 illness, both in the acute and recovery phases.

It is increasingly apparent that nutritional care, including identification of nutritional risk and use of nutrition support, should be a fundamental part of management for these patients. COVID-19 illness negatively impacts nutritional status on many levels; increasing nutritional requirements induced by pyrexia, sepsis, dyspnoea, and reducing nutritional intake due to excessive coughing, dysphagia, dysgeusia, chronic fatigue, poor appetite and food access issues [1,2]. The detection and management of malnutrition in patients with COVID-19 is therefore of fundamental importance.

Given the detrimental effect COVID-19 has on the nutritional status of patients, it is not surprising that, in recent months, clinical nutrition professional organisations across the world have disseminated COVID-19 specific guidelines, consensus opinions, policies and recommendations for clinical practice. In the acute phase and during a hospital admission, oral nutrition support, enteral tube feeding and parenteral feeding regimens are integrated into ward and intensive care unit (ICU) protocols often managed by dietetic and nutritional experts [3,4]. In the community recovery phase, the approach is more varied, coupled with fewer dietitians available in primary care to support these patients.

The aim of this review is to summarise the key themes from the documents that have been published to date that are linked to the nutritional care of individuals with or recovering from COVID-19, including those discharged from hospital and those managed entirely within the community. This summary will support healthcare professionals, informing their clinical practice and highlighting the key areas linked to nutrition support that should be incorporated in primary care COVID-19 recovery pathways.

## 2. Materials and Methods

The search dated from 1 December 2019 to 19 June 2020. Literature was identified by reviewing websites of clinical nutrition professional organisations (those linked to malnutrition, dietetics and nutrition support), following links from these websites to other recommended websites, using website search engines and electronic databases such as PubMed and Cochrane, and using consistent key words throughout, including: COVID-19, nutrition screening, nutrition support (which included enteral nutrition, oral nutrition support, dietary advice, oral nutritional supplements, dietitian), recovery and rehabilitation. Some documents were being regularly updated; this summary is accurate up to 19 June 2020. Dates and version numbers of all publications (where available) were noted.

The inclusion and exclusion criteria are shown in Table 1. In brief, we included COVID-19 specific publications from and/or endorsed by professional organisations with expertise in the field of clinical nutrition. The literature contained guidelines, recommendations, policy/consensus statements or a treatment pathway that were intended for healthcare professionals. Our population was adults with or recovering from a COVID-19 illness in the community (outside of hospital). This included all severities of infection; those who were managed solely in the community to those discharged from a hospital admission, which may or may not have included an ICU admission. The key topic for review was nutrition support in the community, including the continuity of nutritional care from hospital into the community.

Text from each publication from clinical nutrition professional organisations was extracted into tables and reviewed for consistent and recurring themes by authors. Key themes emerged if they were discussed in at least half of the publications; text from the documents was extracted into tables under these key themes. Key messages were summarised from each theme. The themes and key messages were agreed by all authors. Documents from other national organisations (not clinical nutrition specific) that discussed nutrition were also reviewed.

## 3. Results

### 3.1. Summary of Search Findings

Publications were identified that met the inclusion criteria with details summarised in Table 2. We collated fifteen documents specific to COVID-19, nutrition support and recovery in the community, from professional organisations with expertise in the field of clinical nutrition, including: British Dietetic Association (BDA), British Association for Parenteral and Enteral Nutrition (BAPEN), National Nurses Nutrition Group (NNNG), Malnutrition Pathway Group (MPG), European Society for Clinical and Nutrition Metabolism (ESPEN), American Society for Parenteral and Enteral Nutrition (ASPEN), The European Federation of the Associations of Dietitians (EFAD) and the Netherlands National Association of Dietitians (DN), spanning the UK, Europe, and the USA. We recognise that there are other expert nutritional associations, but these represent those that we found to have published in our topic of interest within the search timeframe.

From these publications, we identified 3 main themes, including: malnutrition screening, nutrition support, and continuity of care between settings (hospital discharge into the community) (Section 3.2, Section 3.3 and Section 3.4). A summary of the information from each document supporting the themes and key messages is available in the (supplementary information Table S1).

There were six other national organisations that advocated community nutrition support in COVID-19 specific documents during the review period. Whilst these documents were not from clinical nutrition professional organisations, they acknowledged that nutrition support is required in the recovery phase of COVID-19 illness and we have included a separate, brief overview of these (Section 4).

### 3.2. Screening for Malnutrition Risk in the Community

Of the professional documents reviewed, 60% recommended screening for malnutrition risk [1,2,5,6,7,8,9,12,15]. The BDA, BAPEN and MPG recommended that all individuals with and/or recovering from COVID-19 in the community should have their malnutrition risk assessed at first healthcare professional contact, and subsequently when there is a clinical concern [1,5,12]. ESPEN specified that any patient at risk of poorer outcomes, namely older adults and those with polymorbid conditions, should be screened [2]. The Malnutrition Universal Screening Tool (“MUST”) was one of the screening tools most commonly highlighted [1,2,5,6,7,9,12], especially within the community. “MUST” is a validated screening tool in the community setting [1,2,5,12] for assessing risk of under- and over-nutrition. An online calculator for “MUST” is available on the BAPEN website (www.bapen.org.uk). The “Patients Association Nutrition Checklist” was also cited as useful in identifying nutritional concerns in the older COVID-19 population (www.malnutritiontaskforce.org.uk) [5,6,7,8,12] and ESPEN highlighted tools for nutritional assessment after a positive screening for malnutrition risk such as the Subjective Global Assessment criteria (SGA), the Mini Nutritional Assessment criteria (MNA) or the Global Leadership Initiative on Malnutrition (GLIM) [2]. The documents reviewed indicated that if physical assessments of weight and height are not possible due to COVID-adapted ways of working, recalled and subjective measurements could be used as part of “MUST” [1,5,12]. Use of technology was also indicated to aid remote consultations and support screening [1,6,7,8,9,14,16]. Both BAPEN and the BDA highlighted that virtual consultations can gain useful and valid information to enable screening for malnutrition risk [5,12]. BAPEN offers self-screening using “MUST” as an option for those who are able to, though this does not replace the role of the healthcare professional (HCP) in confirming malnutrition risk or undertaking nutritional assessment (www.malnutritionselfscreening.org) [1,12]. ESPEN, BAPEN and the BDA stated that screening should be completed regardless of a person’s BMI, as obesity can mask malnutrition and muscle wasting (sarcopenia) in patients with COVID-19 [2,6,12]. Importantly, guidelines also emphasised that screening needs to be linked to an appropriate nutrition care plan and with clear documentation [1,6,10,12,15].

*Key Messages:*

*Each patient with or recovering from COVID-19 in the community:*

- *Must be screened for malnutrition with a tool such as “MUST”, upon first contact with a HCP, and when clinically indicated;*
- *Can be screened for malnutrition risk by remote consultation; recalled and subjective measures can be used if necessary with some screening tools, whilst self-screening with a validated tool is also appropriate;*
- *Must have the outcome of their malnutrition screening linked to a documented management plan appropriate for their level of risk, with review plans included.*

### 3.3. Nutrition Support in the Community

#### 3.3.1. Dietary Advice and Oral Nutritional Supplements

All documents reviewed mentioned the need for nutrition support for patients with or recovering from COVID-19. The BDA, ESPEN, MPG and DN recommended a range of treatment strategies and suggested that a flexible mix of interventions would be required [1,2,3,5,6,15]. Four guidelines referenced the National Institute for Health and Care Excellence (NICE) Clinical Guideline 32 (Nutrition Support for Adults: oral nutrition support, enteral tube feeding and parenteral nutrition) and/or NICE Quality Standard 24 (Nutrition Support in Adults) [1,5,6,13], suggesting that NICE nutrition support guidance [17,18] remains relevant for this patient group.

Seven documents (47%) captured the role of dietary advice to optimise dietary intake and in some cases offered practical suggestions for meals and snacks, and for dealing with specific COVID-19 symptoms such as fatigue, nausea, or loss of taste [1,2,3,5,6,14,15]. It was outlined that dietary advice could be verbal including via remote consultation if required or via written patient information sheets [1,3,5], which can be found on multiple websites (e.g., Malnutrition Pathway https://www.malnutritionpathway.co.uk/covid19-resourcetool; BDA https://www.bda.uk.com/resource). Food fortification (a strategy to maximise the nutritional content of foods) was mentioned by some organisations, however it was generally reiterated that care should be taken to focus not just on increasing energy (calories) but to also ensure adequate protein, vitamin and mineral intakes are achieved [1,2,5,6]. Protein requirements can be higher in those recovering from illness, and in individuals with sarcopenia, which is relevant to COVID-19 [1,3]. Some documents highlighted that protein intake needs to be maximised, and ideally should be spread across the day and included in all meals and snacks [1,3,5,15]. In a few instances, recommendations for use of over the counter nutritional supplements, which can be purchased from supermarkets, pharmacies or online to assist in fortifying the diet, were made [1,5].

Many of the documents (11/15; 73%) reviewed mentioned the use of oral nutritional supplements (ONS) in patients with or recovering from COVID-19 in the community [1,2,3,5,6,7,8,9,10,12,15]. Overall, it was recommended that ONS should be considered in line with local policy, when dietary advice alone is insufficient to meet nutritional requirements, or patients are malnourished [1,2,3,5,7,9,10]. From a UK perspective, guidelines indicated that prescribing should meet ACBS (Advisory Committee on Borderline Substances) indications such as disease related malnutrition [1,5,7,8,9]. The MPG highlighted that ONS should be used in addition to a fortified diet and not as a meal replacement [1]. ESPEN offered a prescriptive indication stating that “ONS should be used whenever possible to meet patient’s needs, when dietary counselling and food fortification are not sufficient to increase dietary intake and reach nutritional goals, ONS shall provide at least 400 kcal/day including 30 g or more of protein/day and shall be continued for at least one month” [2]. The MPG also gave guidance on prescription of ONS for those at high risk of malnutrition with COVID-19, recommending the prescription of two ONS per day (600 kcal) for 4 weeks if acutely ill or recently discharged from hospital with COVID-19, or for 12 weeks for those who are chronically ill [1]. The MPG also recommended that the prescription should be tailored to flavour preferences and consider the patient’s physical function [1].

The publications reviewed also offered advice on different aspects of ONS. For example, high protein ONS may be beneficial in certain groups with COVID-19, such as older patients, those with chronic conditions or those who have been discharged from the ICU [1,3,10]; smaller volume (more energy and nutrient dense) ONS were recommended for those struggling with large volumes [1,5,10]; for powdered ONS, as they require the ability to mix and the availability of other ingredients (e.g. milk), it was recommended that consideration needed to be given to the convenience of such a format during the pandemic [1,5,10], with some documents highlighting patient suitability questions [1,10].

#### 3.3.2. Micronutrients

For COVID-19 patients, some documents noted how essential it is to ensure micronutrient intakes are sufficient [1,2,3,5,14,15]. Specifically, ESPEN and DN recommended malnourished patients with COVID-19 consume 100% of the recommended daily intake (RDA) unless there are identified deficiencies, when higher intakes would be required [2,15]. In those unlikely to reach this due to poor dietary intake, one guideline suggested that over-the-counter once daily multivitamin and mineral supplements could be useful in the recovery phase [5]. Vitamin D was the micronutrient most frequently discussed in the guidelines reviewed. Guidance from many organisations, including the BDA, MPG, EFAD, ESPEN and DN, highlighted that vitamin D requires specific attention [1,2,3,5,14,15], particularly in adult patients with limited time outside, for whom it is recommended to consume 10 micrograms (400 International units) per day [1,5]. This is in line with a recent rapid evidence summary looking at Vitamin D and COVID-19 issued by NICE [19]; however, it should be noted that this is an area of emerging evidence.

#### 3.3.3. Tube Feeding in the Community

Upon hospital discharge, it appears uncommon for enteral tube feeding to continue in patients with COVID-19 into the community unless there is dysphagia or neurological dysfunction [3]. The NNG and ASPEN detailed some of the logistics involved in discharging a patient home on an enteral feed [11,16]. Most of the guidelines within our search timeframe were about the safety and practice of different methods of tube placement (nasogastric, gastrostomy, categorisation as aerosol generating procedures) in patients with COVID-19 and these were beyond the scope of this review. Publications of guidance about the use of tube feeding in hospital (including critical illness) were also beyond the scope of this review.

#### 3.3.4. Dietitians and Allied Health Professionals

Specifically, most documents (12/15; 80%) highlighted the role of dietitians in the management of patients with COVID-19 [1,2,3,5,6,7,8,9,10,14,15,16]. Dietitians are clinical nutrition experts and can assist with complex nutritional needs after a referral in line with local policy [6,14]. One guideline by the BDA stated that dietetic referral is recommended for any individual with malnutrition or sarcopenia [5]. ESPEN stated that malnourished individuals should ideally see an experienced professional (e.g., dietitian, clinical nutritionist, specialised physician) [2] while others stated that first line support from another HCP is appropriate, as long as there is an agreed pathway for dietetic referral if progress with nutritional goals is poor or for those with more complex nutritional needs [1,6]. Commonly, guidelines stated that individuals with COVID-19 who also have special dietary requirements (e.g., diabetes, ICU-acquired weakness) or are receiving enteral tube feeding or parenteral nutrition, should be referred to a dietitian [1,5,6]. For individuals that suffer with eating disorders and are adversely affected, support may be required from a specialist mental health dietitian [14]. For the care of those with COVID-19, it was consistently recommended that dietitians should work as part of a multi-disciplinary rehabilitation service including support from, but not limited to, Speech and Language Therapy (SLT), Physiotherapy and Occupational Therapy [1,5,6,7,15]. For example, if individuals have an ongoing issue of dysphagia, a referral should be made to SLT [1]. Of note, some guidelines stated that many individuals recovering from COVID-19 would require physical rehabilitation to regain muscle mass/strength and nutrition support should be aligned with this to aid recovery [3,5,6,14,15].

#### 3.3.5. Community Nutritional Care Pathways

Our review found one national pathway for managing malnutrition in patients with COVID-19 in the community [1] from which local pathways could be developed. Indeed, local COVID-19 pathways that integrate nutrition support can assist healthcare professionals in implementing the oral nutrition support strategies previously described including when to refer to a dietitian or utilise other nutritional support options [1].

As in other patient groups, the guidelines recommended that all nutrition support strategies require a plan, regular review and monitoring [1,5,6,10,12,15]. Patient centred goals should be discussed, and reviewed based on clinical judgement [1].

*Key Messages:*

- *Nutrition support should be considered for individuals with COVID-19 unable to meet nutritional requirements and those who are malnourished. This includes the full range of strategies including dietary advice, ONS and tube feeding.*
- *Protein requirements can be higher in those recovering from COVID-19 illness and with sarcopenia; nutrition support strategies should consider this.*
- *When the diet, including food fortification, is unable to meet nutritional requirements, and/or patients are at high risk of malnutrition, ONS may be required, for a minimum of 4 weeks. The type of ONS and presentation (ready to drink or powdered) should be considered within the context of the specific needs of the individual patient.*
- *Individuals with specialist dietary requirements, or complex needs, should be referred to a dietitian.*
- *Nutrition support should be integrated into care pathways and multi-disciplinary rehabilitation services.*
- *All nutrition support plans should include regular review and monitoring.*

### 3.4. Continuity of Care between Settings (Hospital Discharge into the Community)

The majority (13/15; 87%) of the documents reviewed mentioned some aspect of the discharge process from an acute to community setting for patients with COVID-19 [1,2,3,5,6,7,8,9,10,11,12,14,15]. Some touched on it [8,9,14] while others, such as the BDA, dedicate a specific document to this process [6]. The guidance available typically highlights that the transition from acute to community is a critical step in ensuring patients with COVID-19 who are at risk of malnutrition are identified and referred for appropriate community support. Guidance from the BDA and the MPG mentions patients who have been hospitalised with COVID-19 will be at increased risk of malnutrition and likely to have suffered sometimes significant weight and muscle loss during their stay [1,3,6]. The reasons stated for this include nutritional requirements remaining high in the initial weeks and months post discharge, alongside a reduced dietary intake due to COVID-19 symptoms [1,2,3,6,12]. Documents consistently stated that a clear pathway from acute to community teams needs to be identified, and requires accessible and often rapid communication [1,3,5,6,7,8,11,15]. Furthermore, the publications reviewed typically stated that a documented nutritional discharge plan is essential, which includes malnutrition screening risk, regardless of a person’s body mass index (BMI) [2,6] and a nutrition support plan [5,6,7,8,9,12]. “MUST” is one tool described as being suitable for malnutrition screening in patients with COVID-19 both in the acute and community settings [1,5,12]. “MUST” was designed and validated for both hospital and community use to support continuity of care across settings and, for reasons discussed in Section 3.2 (alternative and subjective measures and a self-screening version), using this tool can help to overcome some of the challenges with screening due to infection prevention measures necessary during the pandemic.

Identification and management of frailty and ICU-acquired weakness (also referred to as Post ICU Syndrome) between settings in patients with COVID-19 was also commonly described. It was stated to occur in nearly half (46%) of patients, lasting up to 2 years post discharge with significant impact on nutritional status and recovery [1,2,15]. The CCSG recommended the assessment of muscle mass or function by tools such as grip strength or 6-minute walk test [3].

*Key Messages:*

- *Effective clear and rapid communication about nutrition, between settings, is essential for continuity of care.*
- *Each patient being discharged from hospital recovering from COVID-19 must have a hospital discharge plan which includes:*

*-A malnutrition risk score using a validated tool such as “MUST”;*
*-Details of nutrition care plans, as nutritional treatment should continue after discharge;*
*-Details of any ICU admission (if relevant).*

## 4. Supporting Publications from Other National and International Organisations

At the time of review, many other organisations (not specific to the field of clinical nutrition), including the WHO and NHS England, had published guidance on the pathways of recovery for individuals with and recovering from COVID-19 illness that included reference to the need for nutritional support. Table 3 details organisations that issued guidance which included some aspect of nutritional care or highlighted the importance of considering nutrition and nutrition support for these patients. The key message from these documents was linked to referral for dietetic support in specific areas of recovery in which nutritional support is required, with one document being endorsed by the BDA [20]. In particular, the publication from the NHS highlighted the importance of individualised nutrition support and dietitians, the role of dietary advice and use of ONS, and the increased need for dietetic expertise to support community enteral tube feeding in light of early supported discharge during the COVID-19 pandemic [21].

## 5. Discussion

Our review assessed relevant guidance for health care professionals around nutritional care for community dwelling adults with and recovering from COVID-19, which were publically available from 1 December 2019 until 19 June 2020. Most guidance was produced in March (27%) and May (47%) with a total of fifteen relevant documents identified. Professional organisations with expertise in clinical nutrition responded in a very short time period to produce guidance. It was also encouraging to see nutrition considered in several other documents relating to care of COVID-19 patients produced by other health-related organisations, highlighting the importance of nutritional care in the recovery from illness.

There were three consistent themes identified in the publications reviewed: (1) screening for malnutrition risk, (2) provision of nutrition support with a documented care plan, and (3) appropriate and effective communication between hospital and community services to support continuation of nutritional care for patients recovering from COVID-19 in the community. It is interesting to note that these themes reflect NICE guidance for the nutritional care of adults: screening for malnutrition risk, provision of appropriate care plans and effective multidisciplinary working across care settings [17]. Therefore, guidance for nutritional care of those recovering from COVID-19 in the community is in principle the same as for any other individual recovering from disease or illness. However, the pandemic brings significant new challenges impacting the ease with which implementation of this guidance can be made. Infection prevention measures have resulted in the need for virtual consultations with increased reliance on subjective or self-reported assessments. This creates particular difficulties for those undertaking nutritional assessments and advising on individualised nutritional care. Some of the guidance documents acknowledge these new challenges and offer practical advice on screening patients for malnutrition risk during virtual appointments and use of subjective markers of nutritional state [1,5,12]. The efficacy of virtual nutritional screening and assessment and impact of virtual care planning on nutritional outcomes is no doubt an issue requiring further investigation to provide evidence for practice.

It is important to remember that this is a new and emerging clinical area with evidence relating to nutritional care of COVID-19 patients limited at the time of this review. Most guidance to date is based on consensus of experience of treating COVID-19 patients used in conjunction with evidence from other relevant clinical conditions or treatments. We anticipate new evidence coming to light in relation to the nutritional care of patients recovering from COVID-19, both in terms of local practice and research publications. We note there is emerging evidence for the use of Vitamin D; most recently, a pilot study from Castillo et al concluded that a high dose of calcifediol in patients acutely unwell with COVID-19 reduced the severity of the disease [25]. Consequently, it is likely that guidelines will be reviewed and updated to incorporate new evidence as and when appropriate. This may include more specific guidance around the most optimal nutritional care to promote the best clinical outcomes, types of enteral feed or ONS, duration of nutrition support, and specific advice for patients of different ages, co-morbidities or treatments for COVID-19.

Our review identified one national pathway for managing malnutrition in UK patients with COVID-19, which was professionally endorsed by the BDA, BAPEN and RCN [1]. The pathway is based on clinical consensus, evidence, and best practice and could be embedded into COVID-19 rehabilitation pathways or used to support the development of local COVID-19 nutritional pathways of care. The extent to which current guidance, highlighted in this review, is integrated into clinical practice and pathways of care still needs to be assessed, alongside exploration of the optimal nutritional management of this patient group. Variability across and between countries in nutritional recommendations for COVID-19 patients was identified, reflecting differences in health care systems, use and access to specific ONS or enteral tube feeds and availability of dietitians. Nevertheless, incorporating the best evidence for nutrition into community-based recovery pathways is vitally important and would benefit from practical evaluations of effectiveness.

It is relevant to note that we did not specifically review patient literature around nutritional care for those with and recovering from COVID-19. The COVID-19 pathway designed for HCPs does include such patient materials, offering coloured leaflets for each level of malnutrition risk according to “MUST” (https://www.malnutritionpathway.co.uk/covid19-resourcetool) [1]. The BDA has also published literature for patients (https://www.bda.uk.com/resource/covid-19-corona-virus-advice-for-the-general-public.html), and NHS England has recently launched a website and mobile application supporting patients in their recovery from COVID-19, which includes a section on nutritional care (https://www.yourcovidrecovery.nhs.uk/). It is of interest that patient literature appears to mirror the themes from our review, reiterating the importance of identifying malnutrition risk with the opportunity for patients self-screening, as well as the role of food fortification and use of ONS.

Due to the nature of the topic, we focussed our review on guidance produced by professional organisations with expertise in clinical nutrition, although parenteral nutrition and guidance for paediatric patients was excluded. We recognise that although our literature search was extensive, it was not a rigorous systematic review; but, with many patients now in the recovery phase of COVID 19, this provides a real insight into the advice available for their nutritional care. We do however appreciate that, due to the nature of the evolving literature in this area, there continues to be publications since our cut-off date.

We hope the themes and key messages from this review will be considered by all community health professionals and included into COVID-19 recovery pathways, ensuring nutritional screening for malnutrition risk, nutritional assessment and appropriate care plans for nutrition support is embedded as a fundamental and integral part of patient care.

## Figures and Tables

**Table 1:** Summary of inclusion and exclusion criteria.

Selection Criteria	Inclusion Criteria	Exclusion Criteria
Publication	- Health care professional organisation (i.e., society, institute, association, Royal College) at a national level with expertise in clinical nutrition - Specialist group affiliated to a professional organisation at a national level - External publication endorsed by a professional organisation - Guidelines, review, consensus of opinion, pathway, recommendations, policy, position statement - English language	- Publication written by an author or a group of authors that is not attributable to a specific professional organisation - Local area, site-specific publications - Single expert opinion or editorial - Webinar, online education session, slide-deck, social media blog/vlog, podcast - Patient resources (patient information sheet) - Non-English language
Population	- Adults (≥18 years of age)	- Children
	- COVID-19 illness/SARS-CoV-2 infection	- Healthy adults or those with non-COVID-19 illness
	- Community based (e.g. outside of hospital, nursing homes, own homes) - Discharge into community	- In an acute setting (e.g., hospitalised) - Patients established on home enteral nutrition (HEN) or parenteral nutrition (PN)
Topic	Malnutrition/screening Nutrition support Nutrition management Dietitian/Dietetic Input Rehabilitation Recovery	Economic impact Public Health Policy Preventative nutrition Feeding devices/insertion Parenteral Nutrition

**Table 2:** A summary of the publications included in this review.

Organisation	Country	Title of Publication	Type of Publication	Date
British Dietetic Association (BDA)	UK			
Optimising Nutrition Prescribing Specialist Group		Community Guidance-MDT (please note this has since changed title) [5]	Guidance	15 June 2020
Education, Professional Development/Policy Team		Nutrition and the COVID-19 discharge pathway [6]	Guidance	19 May 2020
Older People Specialist Group		COVID-19-Recommendations for community action by dietitians for older and vulnerable people living in their own home [7]	Recommendations	25 March 2020
Older People Specialist Group		COVID-19-Recommendations for action by dietitians supporting care agencies working in older people’s own homes [8]	Recommendations	25 March 2020
Older People Specialist Group		COVID-19-Recommendations for community action by dietitians supporting care homes [9]	Recommendations	25 March 2020
Optimising Nutrition Prescribing Specialist Group		Top tips for prescribing oral nutritional supplements and enteral feeds in the community for adults and paediatrics [10]	Recommendations	4 May 2020
Critical Care Specialist Group		Guidance on the management of nutrition and dietetic services during the COVID-19 pandemic [3]	Guidelines/Recommendations	11 May 2020
National Nurses Nutrition Group (NNG) Supported by the Parenteral and Enteral Nutrition Specialist Group (PENG) of the BDA	UK	Practical advice and guidance for management of nutritional support during COVID-19 [11]	Guidelines	April 2020
British Association for Parenteral and Enteral Nutrition (BAPEN)	UK			
Malnutrition Action Group (MAG)		Practical guidance for using MUST to identify malnutrition during the COVID-19 pandemic. Malnutrition Action Group update [12]	Guidelines	May 2020
Nasogastric Tube Safety Special Interest Group		Enteral tube feeding safety in COVID-19 patients (Updated 13 May 2020) [13]	Guidelines	13 May 2020
Malnutrition Pathway Group (MPG) Endorsed by the BDA, BAPEN, Royal College of Nursing (RCN)	UK	A community healthcare professional guide to the nutritional management of patients during and after COVID-19 illness [1]	Guidance	June 2020
European Society for Clinical Nutrition and Metabolism (ESPEN)	Europe	ESPEN expert statements and practical guidance for nutritional management of individuals with SARS-CoV-2 infection [2]	Consensus Statement	31 March 2020
The European Federation of the Associations of Dietitians (EFAD)	Europe	Role of Dietitians in the fight against COVID-19 [14]	Briefing Paper	May 2020
Diëtheek Nederland (Netherlands National Association of Dietitians) (DN)	NL	Nutritional guidance during recovery from COVID-19 (Published in Dutch and English) [15]	Guidelines/Pathway	May 2020
American Society for Parenteral and Enteral Nutrition (ASPEN)	USA	ASPEN report on Nutrition Support Practice Processes with COVID-19: the first response [16]	Report	5 June 2020

**Table 3:** Supporting publications, referring to nutrition support, from other National and International Organisations.

Organisation	Country	Title of Publication	Nutrition Support
National Health Service (NHS England)	England	After-care needs of inpatients recovering from COVID-19 (3rd August 2020) V2 [21]	The document describes the expected immediate and longer-term health needs of COVID-19 patients, following discharge from hospital (whether or not they received intensive care) into home and community settings, following an episode of COVID-19. This has a detailed section on Diet and Nutrition and includes dietetic support in areas including: Tracheostomy; Dysphagia; Cardiac Rehabilitation; Hospital-acquired weakness; Diabetes; Pressure Ulcers and Fatigue. Key messages include: Nutrition is a vital part of the recovery process for all patients with COVID-19. These patients require individually tailored nutrition support. Nutritional rehabilitation needs to be central to community management pathways post-hospital discharge to ensure efficient and effective recovery and to reduce the risk of hospital re-admissions. Although diet enrichment should suffice for most, there seems to be an increased need to use oral nutritional supplements alongside this in malnourished patients to achieve measurable improvements. There may also be some increased need for dietetic expertise to support community enteral tube feeding in light of early supported discharge, to manage dysphagia as a result of ventilation and a need to facilitate resolution of eating while treating malnutrition.
National Health Service (NHS England)	England	COVID-19 prioritisation within community health services (19th March 2020) [22]	Discusses some aspects of therapy interventions that require dietetics support such as malnutrition, frailty and issues of weight management and obesity. Dietetics support for people with significant malnutrition and increased risk of frailty and functional disability.
Faculty of Intensive Care Medicine (FICM) and ICU Steps	England	Recovery and rehabilitation for Patients following the pandemic (May 2020) [23]	Position statement and provisional guidance for the pathway from critical care to recovery in the community. The BDA was not consulted on this document (but will be on the final). Recognise that a Dietitian is part of the rehabilitation team, specifically for: GI-swallow impairment, nutritional/weight loss assessment; Neurological-strokes, extreme fatigue; Skin/soft tissue-pressure sores; Underlying condition management-diabetes, weight management.
World Health Organisation (WHO)	World	Clinical Management of COVID-19 (27th May 2020) [4]	Nutrition mentioned in relation to rehabilitation and malnutrition in relation to older persons. Older person section: Physiological changes with age lead to declines in intrinsic capacity such as malnutrition, cognitive decline, depressive symptoms, and those conditions interact at several levels. These interactions require an integrated approach to the screening, assessment and management of older people.
British Society of Rehabilitation Medicine	UK	Rehabilitation in the wake of COVID-19—A phoenix from the ashes (11th May 2020) Issue 2 [24]	Rehabilitation post ICU -Critical care, acute medical and specialist rehabilitation teams should work closely together to develop rehabilitation pathways for patients who are recovering following treatment in intensive care and high dependency care (whether for Covid-related illness or other critical conditions). Specialist rehabilitation should be delivered by coordinated multi-disciplinary team (includes dietitian).
British Society of Rehabilitation Medicine and Intensive Care Society	UK	Responding to COVID-19 and beyond: A framework for assessing early rehabilitation needs following treatment in intensive care. (24th June 2020) V 1 [20]	This document is endorsed by the BDA (as it was published after our cut-off date, it was not included in the main review). Rehabilitation post ICU Nutritional compromise due to: Disease symptoms: anosmia with or without taste changes, loss of appetite, diarrhoea, nausea and/or vomiting. Clinical course during ICU: (causing muscle wasting or feeding difficulties) hyper-inflammation, the requirement for high levels of sedation, paralysis and proning, prolonged endotracheal intubation on upper aerodigestive tract disuse. ICU-acquired: dysphagia, delirium, weakness, breathlessness and the environment (staff in PPE, cutlery and crockery, upper limb weakness, specific food items and absence of family members). The Rehabilitation Prescription identifies each individual’s need for rehabilitation and specifies how these will be met after discharge.

## Supplementary Materials

The following are available online at https://www.mdpi.com/2072-6643/12/11/3230/s1, Table S1: Text extracted from the guidance documents to support the themes and key messages.
