# Supplementary material for: A Review of Nutrition Support Guidelines for Individuals with or Recovering from COVID-19 in the Community
Source: Nutrients. 2020 Oct 22;12(11):3230. doi: 10.3390/nu12113230 (PMC7690369; doi:10.3390/nu12113230)
Supplement: Supplementary file 1 [file nutrients-12-03230-s001.pdf]

Supplementary Table S1: Text extracted from the guidance documents to support the themes and key messages

Malnutrition Screening in the Community*:

**When and who is screened**
•Screening for malnutrition across all settings, including the community, in patients with and recovering from COVID-19 is key to maximise recovery from the illness<br>•As malnutrition is not only defined by low body mass…persons with obesity should be screened and investigated according to the same criteria [1]
•Patients at risk for poor outcomes and higher mortality following infection with SARS-COV-2, namely older adults and polymorbid individuals, should be checked for malnutrition through screening and assessment. The check should initially comprise the MUST criteria or, for hospitalized patients, the NRS-2002 criteria [2]
•Nutritional risk should be assessed on first contact and when there is concern [3]
•Due to the variety of potential nutritional issues patients with COVID-19 may face, whether in hospital or at home, we recommend routine identification of malnutrition<br>•Malnutrition screening with 'MUST' (Malnutrition Universal Screening Tool) should be undertaken at the earliest opportunity, including… discharge from hospital …it also includes identification of those who are at risk of malnutrition from unplanned weight loss (whether or not they are obese) [4]
•Encourage continued use of nutritional screening e.g. Nutrition Checklist where possible<br>• Identify non face-to-face tools which can support care homes to continue nutrition and hydration care, support and delivery [5]
• Identify non face-to-face tools which can support care staff agency staff to continue nutrition and hydration care, support and [6]
•Identify who will be able to provide nutritional screening for older & vulnerable people in the community<br>• Discuss with your organisation how to support individuals unable to access/interact with technology / telephone consultations [7]
•Every COVID-19 inpatient, regardless of Body Mass Index (BMI), should undergo nutritional screening<br>•As patients with COVID-19 are at a high risk of malnutrition, all should be screened for malnutrition prior to discharge [9]
**Screening**
•Use of a validated screening tool such as 'MUST' is usually recommended<br>•Where it is not possible to obtain physical or self-reported measures of weight or height there are a series of subjective criteria that can be used to form a clinical impression of an individual's malnutrition risk category<br>•Consider direct to self-screening resources available at malnutritionselfscreening.org<br>•During the COVID-19 pandemic, healthcare professionals have had to radically change their way of working, in many cases moving to remote consultations [1]
• Patients at risk of poorer outcomes…should be checked for malnutrition…the check should initially comprise the MUST criteria [2]
•Encourage continued use of 'MUST' nutritional screening where possible [6]
•Consider an assessment of nutritional status/ muscle mass or function (e.g. grip strength or 6-minute walk test) and where possible compare with baseline data from their hospital stay [10]
•Malnutrition can result in sarcopenia….In practice it is important to be aware of both, and moreover be critical in the assessment and diagnosis of both [8]
•The Malnutrition Universal Screening Tool (MUST) is a validated tool to screen for malnutrition across all care settings<br>•Consider using the subjective measures of 'MUST' or Patients Association Nutrition Checklist can be used instead to help identify risk of malnutrition when conducting virtual reviews. Both are validated and can be used instead to help<br>identify risk of malnutrition when conducting virtual reviews [3]

**The text is cited verbatim from the corresponding reference*

•Is 'MUST' still a feasible option for the MDT to use or is the nutrition checklist or is a simpler option at this time? [7]
•Encourage continued use of nutritional screening e.g. Nutrition Checklist where possible [5]
•Where it is not possible to obtain physical or self-reported measures of weight or height there are a series of subjective criteria that can be used to help form an overall clinical impression of an individual's malnutrition risk category<br>• Where undertaking virtual consultations, gain reliable, reported values from the patient (or a carer/family member) about weight, height, weight history<br>•Our self-screening website (www.malnutritionselfscreening.org) is available for patients and carers to find out more about how they can identify malnutrition themselves<br>•There is also a Patients Association Nutrition Checklist that may also be helpful [4]
•Further information: MUST; Patient Association- checklist for patients or healthcare [9]
**Link to plan**
•Importantly, make sure screening is linked to an appropriate action plan, as defined by your local policy for the management of malnutrition and specific nutritional support guidance for patients with Covid-19 [4]
•Malnutrition Pathway COVID-19 Illness (Community nutrition support pathway using MUST) [1] (Care plan linked to malnutrition risk including subjective measures)
• Acute and community dietetic teams should work collaboratively and agree patient care pathways within their local area [11]
•First-line dietitians are being expected to support these discharged COVID-19 patients following their hospitalization. It is thus important that there are clear guidelines and a care pathway in place [8]
•(nutrition screening)…. This should be recorded on a discharge documentation and a clear plan put in place to provide nutrition support where needed [9]

Nutrition Support in the Community*:

**Diet Advice / Food Fortification**
•Reasons why COVID-19 can affect dietary intake: Respiratory issues; Change to taste and smell; Temperature and Infection; Fatigue and weakness; Isolation<br>•Making the most of food via food fortification when required protein - which may warrant special attention due to increased needs for protein during illness and recovery<br>•Care should be taken with food-based strategies to ensure adequate provision of protein, vitamins and minerals<br>•A number of nutritional supplements are available for self-purchase in supermarkets, pharmacies and online. Consider how accessible these may be in the COVID-19 pandemic<br>•The severe symptoms and consequences of COVID-19 may exacerbate malnutrition…but may also predispose a patient to malnutrition<br>•The COVID-19 dietary advice leaflets are intended to give general advice on the optimisation of intake [1]
•COVID-19 can be accompanied by nausea, vomiting and diarrhoea impairing food intake and absorption<br>•Subjects with malnutrition should try to optimize their nutritional status, ideally by diet counselling from an experienced professional [2]
•Nutrition-related factors to note may include (COVID-19) symptoms which could impede nutritional intake<br>•Advising on a nutrient dense diet, with particular attention to sufficient protein or extended periods, likely better given as periodic doses<br>•It is suggested that it may require provision of significantly more calories and protein, although further research is needed<br>•CCSG recommends that patients are provided with written literature until dietetic counselling may be appropriate or possible. Local or nationally developed literature can be used [10]
•Consider the following symptoms associated with Covid-19 that could reduce ability to eat and drink for adequate nutritional intake: shortness of breath, persistent coughing, new

**The text is cited verbatim from the corresponding reference*

onset of dysphagia, loss of sense of smell and taste<br>•Food fortification advice should focus on nutrient dense foods and should include good sources of protein as part of all meals and snacks<br>•For patients unable to meet their nutritional needs from their diet and food fortification discuss use of over the counter nutritional supplements<br>•Patients should have written instructions on when, how and for how long to take the product in conjunction with food fortification guidance [3]
•Encourages the consumption of adequate energy, protein and micronutrients…<br>•Community dietetic teams should be able to advise on the best approach for individual patients with a focus on nutrient dense nutrition and not just calories [9]
•Appropriate spreading of nutrition across the day / increase frequency of meals. It is preferred that every meal contains 25g protein<br>•It is recommended to adapt the dietary advice to the following individual wishes and needs (budget, religion, culture, conviction, knowledge)<br>•With sarcopenic obesity: Protein are complete, high quality, and are offered multiple times per day.<br>•Energy and protein rich nutrition (estimated using the WHO formula, the Harris and Benedict formula or measurement) + condition<br>•Protein requirements: 1.2 to 2.0 g/kg actual bodyweight [8]
•Dietitians can recommend nutrient rich, fortified, tasty foods or specialist nutrition supplements to help people regain weight and muscle that may have been lost [12]
**Oral Nutritional Supplements (ONS)**
•ONS may be required in those with a medium or high risk of malnutrition…<br>•ONS should be used in addition to normal diet and not as a food replacement<br>•Ensure ONS prescription requests meet the Advisory Committee on Borderline Substances (ACBS) indications<br>•High protein ONS may be required to achieve this (recommended intake) amongst older patients, those with chronic conditions and those discharged from ICU<br>• Ready-to-drink, high energy, low volume ONS should be considered to reduce time/effort needed to prepare and consume the ONS<br>•High risk MUST score 2 or more; or patient has a reduced appetite / is underweight / feels weak / has a long term health condition – provide dietary advice plus prescribe 2x ONS** per day for 4 weeks (acutely ill / recent hospital discharge) or 12weeks (chronically ill) [** patients with breathlessness may benefit from a compact or low volume supplement] [1]
•Oral nutritional supplements (ONS) should be used whenever possible to meet patient's needs, when dietary counselling and food fortification are not sufficient to increase dietary intake and reach nutritional goals, ONS shall provide at least 400 kcal/day including 30 g or more of protein/day and shall be continued for at least one month. Efficacy and expected benefit of ONS shall be assessed once a month [2]
•Utilising high protein oral nutrition supplements as required [10]
•Prescribed oral nutritional supplements are to be used appropriately, in line with national and local guidance<br>•Lack of financial resources is not an ACBS indication for prescription<br>•ONS should be considered when food intake (including food fortification) does not meet nutritional goals and where the ACBS criteria for prescribing that ONS are met<br>•Prescription of the most cost-effective, clinically appropriate ONS should be requested [3]
•Oral nutritional supplements (ONS) should only be considered where clinically indicated (in line with local NHS guidance) & where people meet ACBS indications (ACBS indications do not include food access issues) [5-7]
•Support may take a number of forms. This may include the ongoing provision of home enteral feeding or oral nutrition support (with or without oral nutrition supplements) [9]
•Work with local Prescribing Support Dietitian/s and/or CCG or Health Board Medicines Management Teams to review current local formulary to see if additional products need to be added that would support rehabilitation of patients suffering/recovering from COVID-19 as patients may require more concentrated ONS that are high in protein<br>•If clinically appropriate, ONS in powder form are the most cost-effective supplement available; however, the following considerations need to be made on suitability:<br>1. Does the patient/carer have the physical ability to make up the powdered ONS as directed on the package?<br>2. Does the patient/carer have access to both a fridge and milk (fresh or UHT)?

**The text is cited verbatim from the corresponding reference*

3. Does the patient have adequate storage for the powder ONS? [11]
•If there is uncertainty about achieving adequate energy and protein intake, start oral nutritional supplements or additional modules or continue use of oral nutritional supplements after hospital discharge [8]
•…action can be put in place including dietary intervention, nutrition support (oral nutritional supplements, enteral tube feeding and parenteral nutrition) and other treatments as required [4]
**Dietitian / Allied Health Professionals**
•For complex patients, those at high risk of malnutrition and those at medium risk of malnutrition who do not improve despite preliminary intervention, consider a dietetic referral<br>•Always use clinical judgement in using the Malnutrition Pathway COIVD-19 resources in those with complex conditions which may require referral to a dietitian for specialist dietary advice<br>•Referral to speech and language therapy should be made for patients with dysphagia or swallowing difficulties [1]
•Subjects with malnutrition should try to optimize their nutritional status, ideally by diet counselling from an experienced professionals (registered dieticians, experienced nutritional scientists, clinical nutritionists and specialized physicians) [2]
•Dietary counselling to include increased physical activity alongside a healthy diet but still sufficient protein alongside exercise [10]
•Patients who are malnourished or at high risk of malnutrition, as well as those with specialist diet requirements, (e.g. diabetes, renal disease) should be referred to a dietitian<br>•Dietitians should work as key MDT members within COVID-19 rehabilitation services…This may include support from physiotherapy and occupational therapy<br>•The combination of well-balanced nutrition and physical activity play a key role in recovery [3]
•Leading therapy pathways should contact local dietetic services to ensure nutrition is embedded within them<br>•Dietitians should guide other HCPs to reliable first line advice and agree referral pathways to support patients with increased or more complex needs<br>•…may also require support from other members of the MDT, including but not limited to: psychological support, occupational therapy and speech and language therapy<br>•To assist recovery, rehabilitation and repletion of muscle mass patients will need ….adequate energy, protein and micronutrients combined with exercise rehabilitation as advised by physiotherapy [9]
•When working remotely, dietitians are expected to conduct nutritional assessments….provide patient and family education and document [13]
• Who would benefit most from dietetic input & advice?<br>• What dietetic leadership & supervision can be provided to the MDT regarding nutrition and hydration advice?<br>•Contact & work in partnership with speech and language therapists for those requiring texture modified diet & fluids<br>•Contact and work in partnership with other colleagues including physiotherapists, occupational therapists and falls teams [7]
•This will often involve …close liaison with other health professions who are supporting rehabilitation<br>•People's mental health will also have been affected by increased isolation and worry, and for those with conditions such as eating disorders, support from specialist mental health dietitians will be vital, especially as many of their support networks may not be available<br>•Dietitians can recommend nutrient rich, fortified, tasty foods or specialist nutrition supplements to help people regain the weight and muscle that may have been lost<br>•Dietitians are using technology to great effect, including video-conferencing and specialist apps to provide consultations and support to patients who need them [12]
• We ask dietitians to consider implementing the following actions to support non-NHS services to care for our high risk population; -Discuss and agree plans for how nutrition support can be provided for those who have contracted COVID-19 [5]
• We ask dietitians to consider implementing the following actions to support community services and care homes to care for our high risk population; -Discuss and agree plans for how nutrition support can be provided during a COVID-19 outbreak within an individual care home<br>• Technology may offer opportunities for dietitians to provide support to patients and colleagues remotely [6]

**The text is cited verbatim from the corresponding reference*

• Agree a referral process to support acute dietetic staff<br>• Acute and community dietetic teams should work collaboratively and agree patient care pathways within their local area [11]
•Dietitians may contact patients by telephone to obtain information for the assessment [13]
•Nutritional support should be offered as indicated in NICE 32, 2017 [14]
•Now that the first COVID-19 patients are starting to be discharged from hospital wards, these patients are being directed towards our first-line dietitians to begin their recovery phase post-hospitalisation.<br>•Discuss recommendations with appropriate physiotherapist and potentially modify according to the intensity of the movements, exercises or trainings [8]
**Micronutrient supplementation**
• Meeting vitamin and mineral requirements during illness and when appetite is poor – supplementation may be required<br>• The importance of vitamin D and supplementation, particularly in those who are spending a lot of time indoors. It is recommended that adults take a supplement containing 400 international units (IU) [10 micrograms] of vitamin D per day [1]
•We suggest the provision of daily allowances for vitamins and trace elements be ensured to all malnourished patients at risk or with COVID-19 [2]
•Assessing the need for additional micronutrient supplementation, including Vitamin D depending on individual circumstances [10]
•Adequate vitamin and mineral intake is also essential but may be difficult to achieve with a reduced food intake<br>•It may be beneficial to advise on an over the counter, once daily multivitamin and mineral supplement during the recovery phase<br>•Ensure the supplement contains 10micrograms of Vitamin D if the patient is unable to go outside<br>•The patient should have a clear documented nutritional care plan which includes goals including<br>when the product will be stopped and efficacy should reviewed regularly (ideally monthly) [3]
•…such as concerns about Vitamin D status amongst those staying indoors for a prolonged period [12]
•Vitamins and minerals: all requirements determined by 100% RDA, unless there are indicated deficiencies or increased requirements.<br>•Vitamin D: recommendations / requirements according to public health recommendations [8]

Continuity of care between settings (hospital discharge into the community) *

**Discharge plan and treatment**
•Nutritional treatment should continue after hospital discharge<br>•Prolonged reported duration of ICU stay above two weeks for many COVID-19 patients is likely to further enhance muscle-catabolic conditions<br>•As malnutrition is not only defined by low body mass…persons with obesity should be screened and investigated according to the same criteria [2]
•Hospital teams discharging patients with identified nutritional concerns should communicate this in writing to primary care teams<br>•The MUST is a validated tool used across care settings<br>•Communication between different health professionals and settings is essential for seamless delivery of care [3]
•The primary (acute) dietitian oversees the post ICU syndrome (PICS) patients if they are transferred from the hospital or other healthcare facility<br>•First-line dietitians are being expected to support these discharged COVID-19 patients following their hospitalization. It is thus important that there are clear guidelines and a care pathway in place [8]

**The text is cited verbatim from the corresponding reference*

•Work with acute staff to ensure nutritional risk scores & nutritional care plans are included on hospital discharge documentation [5] [6]
•Tailored nutritional management is recommended for patients recovering from ICU after hospital discharge<br>•Screening for malnutrition at the point of hospital discharge to assess their ongoing need for nutrition support at home, and review by a dietitian, is recommended<br>•The severe symptoms and consequences of COVID-19 may exacerbate malnutrition…but may also predispose a patient to malnutrition<br>•Use of a validated screening tool such as MUST is usually recommended<br>•Care should be coordinated between the acute and community settings [1]
•Ensure a clear handover to follow-on dietetic service of the nutritional care plan and information on nutrition or functional status<br>•In the initial weeks and months following discharge there may be ongoing weight loss or continued reduced desire to eat [10]
•Every COVID-19 inpatient, regardless of Body Mass Index (BMI), should undergo nutritional screening…this should be recorded on discharge documentation<br>•As patients with COIVD-19 are at a high risk of malnutrition, all should be screened for malnutrition prior to discharge<br>•Dietetic teams should ensure clear communication between acute and community services as part of discharge processes [9]
•We would recommend a nutritional assessment prior to handover with an updated nutritional care plan<br>•Malnutrition screening with 'MUST' (Malnutrition Universal Screening Tool) should be undertaken at the earliest opportunity, including… discharge from hospital …it also includes identification of those who are at risk of malnutrition from unplanned weight loss (whether or not they are obese) [4]
•Identify and contact local hospital discharge teams, community coordination teams and Executive Lead responsible to offer support<br>•Ensure nutritional risk scores & nutrition care plans are included on hospital discharge documentation [7]
•Liaise with your discharge teams, as many have put in place or are considering rapid discharge to community hospitals and nursing homes<br>•If NG or NJ feeding is required for an individual on discharge, it is essential that clear communication and discharge criteria are set [15]
•Even when people are well enough to leave hospital after COVID-19, their journey is not over. Dietitians have an important role to play in rehabilitation, reducing risk of complications and shortening recovery times<br>•Patients who have suffered COVID-19 and been hospitalised will be at increased risk of malnutrition and will likely have suffered loss of muscle during their stay [12]
•Acute staff should inform community colleagues of the nutritional care plan for discharged patients<br>•It would be beneficial for community staff to receive regular, brief updates from the acute setting to understand when COVID-19 patients are starting to transition out into the community and what level of support they will require [11]

**The text is cited verbatim from the corresponding reference*

References

1. Managing Adult Malnutrition. *A community healthcare professional guide to the nutritional management of patients during and after COVID-19 illness*; https://www.malnutritionpathway.co.uk/covid19-community-hcp (accessed on 19 June 2020).
2. Barazzoni, R.; Bischoff, S.C.; Breda, J.; Wickramasinghe, K.; Krznaric, Z.; Nitzan, D.; Pirlich, M.; Singer, P.; endorsed by the, E.C. ESPEN expert statements and practical guidance for nutritional management of individuals with SARS-CoV-2 infection. *Clinical nutrition (Edinburgh, Scotland)* **2020**, *39*, 1631-1638, doi:10.1016/j.clnu.2020.03.022.
3. British Dietetic Association: Optimising Nutrition Prescribing Specialist Group. *Community Guidance-MDT*; https://www.bda.uk.com/resource/nutritional-guidance-for-primary-care-teams.html (accessed on 15 June 2020).
4. British Association of Parenteral and Enteral Nutrition: Malnutrition Advisory Group. *Practical guidance for using 'MUST' to identify malnutrition during the COVID-19 pandemic, Malnutrition Action Group (MAG) update;* https://www.bapen.org.uk/pdfs/covid-19/covid-mag-update-may-2020.pdf (accessed on 30 May 2020).
5. British Dietetic Association Older People Specialist Group. *COVID-19 - Recommendations for community action by dietitians to support care agencies working in older people's own homes;* https://www.bda.uk.com/resource/covid-19-recommendations-for-action-by-dietitians-supporting-care-agencies-working-in-older-people-s-own-homes.html (accessed on 25 March 2020).
6. British Dietetic Association Older People Specialist Group. *COVID-19 - Recommendations for community action by dietitians supporting care homes*; https://www.bda.uk.com/resource/covid-19-recommendations-for-community-action-by-dietitians-supporting-care-homes.html (accessed on 25 March 2020).
7. British Dietetic Association: Older People Specialist Group. *COVID-19- Recommendations for community action by dietitians for older and vulnerable people living in their own home.*; https://www.bda.uk.com/resource/covid-19-recommendations-for-community-action-by-dietitians-for-older-and-vulnerable-people-living-in-their-own-home.html (accessed on 25 March 2020).
8. Dietheek. *Nutritional Guidance During Recovery from COVID-19*; https://european-nutrition.org/wp-content/uploads/2020/05/Nutritional-guidance-during-recovery-from-COVID-19.pdf (accessed on 30 May 2020).
9. British Dietetic Association. *Nutrition and the COVID-19 discharge pathway;* https://www.bda.uk.com/resource/nutrition-and-the-covid-19-discharge-pathway.html (accessed on 19 May 2020).
10. British Dietetic Association: Critical Care Specialist Group. *Guidance on management of nutrition and dietetic services during the COVID-19 pandemic;* https://www.bda.uk.com/resource/critical-care-dietetics-guidance-covid-19.html (accessed on 11 May 2020).
11. British Dietetic Association Optimising Nutrition Prescribing Specialist Group. *Top tips for prescribing oral nutritional supplements and enteral feeds in the community for adults and paediatrics;* https://www.bda.uk.com/resource/top-tips-for-prescribing-oral-nutritional-supplements-and-enteral-feeds-in-the-community-for-adults-and-paediatrics.html (accessed on 4 May 2020).
12. The European Federation of the Association of Dietetics. *Role of Dietitians in the fight against COVID-19. EFAD Briefing Paper*; http://www.efad.org/media/1985/role-of-dietitians-in-the-fight-against-covid19-efad-briefing-paper-may-2020.pdf (accessed on 13 May 2020).
13. Mulherin, D.; Walker, R.; Holcombe, B.; Guenter, P. *ASPEN Report on Nutrition Support Practice Processes with COVID-19: The First Response. ASPEN Clinical Practice Committee*;

**The text is cited verbatim from the corresponding reference*

https://www.nutritioncare.org/uploadedFiles/Documents/Guidelines_and_Clinical_Resources/COVID19/ASPEN%20Clinical%20Report%20on%20Nutrition%20Support%20Practice%20Processes%20with%20COVID-19%205-26-2020.pdf (accessed on 30 May 2020).
14. British Association of Parenteral and Enteral Nutrition: Nasogastric Tube Safety Special Interest Group. *Enteral tube feeding safety in COVID-19 patients (Updated 13/05/2020)*; https://www.bapen.org.uk/pdfs/covid-19/covid-19-and-enteral-tube-feeding-safety-13-05-20.pdf (accessed on 20 May 2020).
15. National Nurses Nutrition Group. *Practical advice and guidance for management of nutritional support during COVID-19. Version 2;* https://www.nnng.org.uk/2020/04/covid-19-statement-and-information/ (accessed on 14 April 2020).

**The text is cited verbatim from the corresponding reference*
